# Fabrication of Magnetic Molecularly Imprinted Beaded Fibers for Rosmarinic Acid

**DOI:** 10.3390/nano10081478

**Published:** 2020-07-28

**Authors:** Engy M. Saad, Nesrine Abdelrehim El Gohary, Basma M. El-Shenawy, Heba Handoussa, Anke Klingner, Mohamed Elwi, Youssef Hamed, Islam S. M. Khalil, Rasha Mohamed El Nashar, Boris Mizaikoff

**Affiliations:** 1 Pharmaceutical Chemistry Department, Faculty of Pharmacy and Biotechnology, German University in Cairo, Cairo 11835, Egypt; Engy.yehia@guc.edu.eg; 2 Pharmaceutical Technology Department, Faculty of Pharmacy and Biotechnology, German University in Cairo, Cairo 11835, Egypt; basma.el-shenawy@guc.edu.eg; 3 Pharmaceutical Biology Department, Faculty of Pharmacy and Biotechnology, German University in Cairo, Cairo 11835, Egypt; heba.handoussa@guc.edu.eg; 4 Physics Department, German University in Cairo, Cairo 11835, Egypt; anke.klingner@guc.edu.eg; 5 Materials Engineering Department, Faculty of engineering and Materials Science, German University in Cairo, Cairo 11835, Egypt; mohamed.elwi@guc.edu.eg; 6 Mechatronics Department, Faculty of engineering and Materials Science, German University in Cairo, Cairo 11835, Egypt; youssef.sabry@student.guc.edu.eg; 7 Department of Biomechanical Engineering, University of Twente, 7522 NB Enschede, The Netherlands; i.s.m.khalil@utwente.nl; 8 Chemistry Department, Faculty of Science, Cairo University, Giza 12613, Egypt; 9 Institute of Analytical and Bioanalytical Chemistry, Ulm University, 89081 Ulm, Germany

**Keywords:** molecularly imprinted fibers, electrospinning, polycaprolactone, design of experiments, microrobots, drug release

## Abstract

The present study describes the fabrication of molecularly imprinted (MI) magnetic beaded fibers using electrospinning. Rosmarinic acid was selected as exemplary yet relevant template during molecular imprinting. A “design of experiments” methodology was used for optimizing the electrospinning process. Four factors, i.e., the concentration of the biodegradable polymer (polycaprolactone), the applied voltage, the flow rate, and the collector distance were varied in a central composite design. The production process was then optimized according to the suitability of the beaded fibers during microrobot fabrication, actuation, and drug release. The optimum average fiber diameter of MI beaded fibers was determined at 857 ± 390 nm with an average number of beads at 0.011 ± 0.002 per µm^2^. In vitro release profiles of the optimized MI beaded fibers revealed a lower burst rate and a more sustained release when compared to control fibers. Magnetic control of the MI beaded fibers was successfully tested by following selected waypoints along a star-shaped predefined trajectory. This study innovatively combines molecular imprinting technology with magnetic microrobots enabling targeted drug delivery systems that offer precise motion control via the magnetic response of microrobots along with selective uptake of a drug into the microrobot using MI beaded fibers in future.

## 1. Introduction

The combination of molecular imprinting, electrospinning, and magnetic nanoparticles for fabricating magnetic molecularly imprinted beaded fibers has great potential for their future use as drug delivery vehicles. 

Molecular imprinting is a technique for synthesizing biomimetic receptors based on the lock-and-key model [1,2], whereby specific molecular recognition sites are introduced into polymeric matrices. These recognition sites exhibit templated and distinctive selectivity towards the targeted molecules [3,4]. Imprinted polymers are commonly synthesized via assembly of template molecules with complementary functional monomers either by reversible covalent or noncovalent interactions. The assembled prepolymerization complex is then copolymerized with suitable cross-linker creating a porous three-dimensional polymer matrix with stereospecific binding cavities complementary in shape, size, and chemical functionality to the template of interest [5,6]. 

Electrospinning is a facile and versatile method for creating polymer-based ultrafine fibers of diameters ranging from the micro- to nanometer scale. The application of ultrafine fibers is of specific interest in drug delivery due to their high loading capacity, high encapsulation efficiency, and effective production cost. Therefore, electrospinning fabrication strategies have gained importance in areas such as drug delivery, wound dressing, and the like [7,8,9,10]. The basic principle of electrospinning involves the application of relatively high voltages for charging a pending droplet of a polymer solution, ejection of polymer jets by electric forces, conversion of the jets into fine fibers by solvent evaporation, and collection of thus produced fibers at the nearest grounded surface producing a randomly oriented web of micro- or nanoscale fibers [11,12]. The formation of bead-like structures is likely observed within such electrospun mats as a by-product of the electrospinning process.

In turn, the actual occurrence of beaded fibers is more likely associated with instabilities of the polymer jet. The surface tension of the polymer solution is the main cause for capillary deformation of electrospinning jet, which then leads to the formation of droplets within the filaments creating stable beads along the string structures. These bead-on-string structures qualify as drug reservoirs with the number of beads ideally tunable to and correlated with the application requirements [9,13]. Furthermore, magnetic particles may be incorporated into the beads, thereby introducing magnetic properties useful in a wide variety of applications including data storage [14], tissue engineering [15], and microrobots with targeted drug delivery potential [16,17]. 

Despite the inherent simplicity, the interaction between different electrospinning processing parameters such as the applied voltage, the flow rate, and the distance between the tip of syringe needle and the collector appears to be complex. In addition, solution parameters including the polymer concentration, its molecular weight, viscosity, conductivity, surface tension, and ambient parameters including temperature and humidity further convolute the variable canvas spanning the process design space. Therefore, it is a time-consuming process if one varies one factor at a time for process optimization [18,19]. Design of experiments (DOE) methodologies such as central composite experimental design provide better control on the variable data space in electrospinning manufacturing techniques, whereby the influence of each parameter, and their potential interactions, are considered via examining different combination of factors at different levels using linear regression analysis and the analysis of variance (ANOVA) as mathematical models. The resulting output provides comprehensive insight on the decisive variables and their potential interactions yielding a linear regression equation to predict the desired fiber morphology within a set of selected parameters [18]. 

The present study aims at developing magnetic electrospun beaded fibers with incorporated MI binding sites, which enable a wide range of application scenarios especially in the healthcare domain. Rosmarinic acid, a naturally occurring phenolic acid, was selected as exemplary yet relevant template during molecular imprinting due to its potent antioxidant activity [20] and potential therapeutic use for various disorders including cancer [21], hepatic diseases [22], and neurodegenerative disorders [23].

During previous studies, it has been demonstrated that electrospun magnetic beaded fiber structures resembling the shape of a sperm could behave as microrobots that are readily controlled using external magnetic fields, and may thus be directed to specific locations, and may thus serve as drug delivery vehicles in future [16,17].

A circumscribed central composite design has been used to examine the interaction of four relevant parameters affecting the electrospinning process, i.e., the polymer concentration, the applied voltage, the collector distance, and the flow rate. Last but not least, the obtained fibers were characterized via SEM, FTIR, and EDX analysis corroborating that the desired design has been successfully achieved.

## 2. Materials and Methods 

### 2.1. Fabrication and Characterization of Magnetic MI Fibers

High-molecular-weight polycaprolactone (PCL) (70,000–90,000 g/mol; Sigma–Aldrich, Steinheim, Germany) was dissolved in a mixed solvent system of dichloromethane (DCM) (Sigma–Aldrich, Steinheim, Germany) and tetrahydrofuran (THF) (Sigma–Aldrich, Steinheim, Germany) in a (1:1 *v*/*v*), by stirring at room temperature. Rosmarinic acid (RA) (Sigma–Aldrich, Steinheim, Germany) was dissolved in 0.5 mL ethanol (Sigma–Aldrich, Steinheim, Germany), whereas polyallylamine (PAM) (Alfa Aesar, Karlsruhe, Germany) was dissolved in 0.5 mL water; the two solutions were mixed together for 15 min by stirring to allow the assembly of RA with PAM. The solution mixture was slowly added to PCL solution under stirring for 1 h. Prior to electrospinning, magnetite iron (II and III oxide) (50–100 nm; Sigma–Aldrich, Steinheim, Germany) was added as 60% (*w*/*w*) of PCL present and was thoroughly mixed.

A vertical electrospinning setup was used to fabricate fibers mats. The prepared magnetic surfactant-free microemulsions (SFME) were filled in a 5 mL sterile syringe fitted with a needle 22G x 1 ¼” connected to positive terminal of high-voltage power supply (Leybold, Cologne, Germany). SFME were ejected by a syringe pump (CMA 402; Harvard apparatus, Holliston, MA, USA). The electric field was created by high voltage applied between the needle tip of the syringe and the collector. Fibers were formed and accumulated on plastic petri dishes placed on the surface of a stationary metal collector.

Morphological examination of the electrospun mats was done using SEM (Quanta 250 FEG SEM; Thermo Scientific, Waltham, MA, USA) at different magnifications. The samples were subjected to gold sputter coating prior to examination to prevent surface charging under the electron beam. Fiber diameter (FD), number of beads present per µm^2^ (NB), and bead diameter (BD) measured as bead width perpendicular to the fiber axis were analyzed using “Image J” software (version 2.35 for Windows, 64 bit, National Institutes of Health, Bethesda, MD, USA) at 4000× magnification. Diameters of clearly visible fibers and beads were measured and the average FD and BD were calculated using the data collected from each image. Beads per image were counted, and average NB was calculated. 

### 2.2. Design of Experiment Methodology

**Figure 1:**
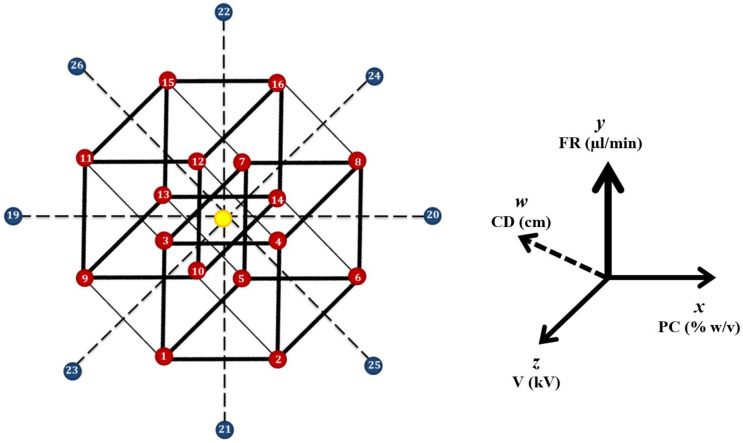
Schematic illustration of the design space of experiments as a function of *x* (polymer concentration), *y* (flow rate), *z* (applied voltage), and *w* (collector distance). The red points represent the 16 cube points (runs 1–16), the blue points represent the 8 axial points (runs 19–26), and the yellow point represents the center point’s replicates (runs 17, 18, 27, and 28).

The preparation of magnetic MI beaded fibers was optimized via a design of experiments (DOE) methodology, in which Minitab^®^ 17 software (version 17.1.0, Minitab Ltd., Coventry, Warwickshire, United Kingdom) was utilized for the statistical analysis and modeling. Four factors; polymer concentration (PC) (4–16% *w*/*v*), collector distance (CD) (1–17 cm), flow rate (FR) (8–20 µL/min), and applied voltage (V) (13–25 kV), having reported effect on the electrospinning process [24,25], were varied in a circumscribed central composite experimental design. A circumscribed design was implemented with 5 factor levels set for each of the 4 factors. The design was composed of 28 randomized runs (summarized in Supplementary Table S1), according to *N* = 2*^f^* + 2*f* + *n_c_*, where *f* is the number of factors and *n_c_* is center point replicates number. Therefore, the design was classified into 16 cube points, 8 axial points, and 4 center points, as illustrated in Figure 1. To avoid any error that might be introduced into the current study due to changes in ambient conditions, trials were split into two blocks, factorial block and axial block with two center points’ runs assigned to each block. The experiments were run on 2 consecutive days with average temperature of 25 °C and average humidity of 57%. Higher temperatures were avoided to prevent the production of fibers with smaller diameters due to decrease in the solution viscosity. Low humidity was avoided to prevent rapid solvent evaporation and drying, which could lead to clogging of the needle tip [26]. 

Regression analysis was performed to describe the relationship between the 3 selected responses, i.e., average FD, NB, and BD, and the four electrospinning parameters. Stepwise regression using the backward elimination method was utilized to obtain the regression equations, whereby all potential terms, i.e., linear, quadratic, and cubic, and their 2- and 3-way interactions were initially added to the model with the least significant terms sequentially removed until all model variables achieved *p*-values ≤ 0.05. Transformation of response variables was examined to enhance model fitting. Adjusted and predicted *R*^2^ values were computed and residual analysis was performed to verify the goodness of model fit and predictability.

The predictive capabilities of the regression models were tested by performing three individual electrospinning runs using a random combination of factor settings within the model limits. SEM images were collected and average FD, NB, and BD were determined and calculated for each run at 4000× magnification. The prediction interval for each run was displayed with a 95% confidence level, and the regression models were expected to predict the new observations within these limits.

### 2.3. Optimization of Fiber Fabrication

The optimal combination of electrospinning parameters was identified to obtain the desired morphological features enabling the production of magnetic beaded fibers. Magnetic MI beaded fibers were then prepared using the obtained optimized polymer concentration, collector distance, flow rate, and applied voltage settings. Control magnetic beaded fibers loaded with RA were prepared using the same composition and factor settings as a control, yet in the absence of PAM. 

### 2.4. Characterization of Optimized MI Fibers

IR spectra of MI and control fibers were recorded in the range of 4000–400 cm^−1^ using a Nicolet Avatar 380 spectrometer (Thermo Fisher Scientific, Munich, Germany). Energy-dispersive X-ray (EDX) analysis was performed to characterize the heads and tails of MI beaded. The EDX system was coupled with SEM.

### 2.5. Drug Encapsulation and In Vitro Release Studies

Five mg of the desired MI and control loaded beaded fibers were weighed, and completely dissolved in 1 mL formic acid; this was followed by the addition of 4 mL water to precipitate PCL. The samples were centrifuged for 45 min at 13,000 rpm to sediment magnetite particles and PCL present, then filtered and diluted 10 times before determining the amount of encapsulated RA. All chromatographic measurements were performed using an ACQUITY H-Class preparative HPLC System (Waters, Milford, MA, USA) at wavelength of 330 nm. The chromatographic column used was BDS Hypersil C18 (150 × 4.6 mm, 5 µm) (Thermo Scientific, Waltham, MA, USA). The mobile phase consisted of 0.1% phosphoric acid in water (A) and 0.1% phosphoric acid in methanol (B). The gradient used was linear gradient from 40% to 50% (B), 0–10 min [27]. The flow rate was 1 mL/min. Detection was carried out at a wavelength of 330 nm, with an injection volume of 10 μL. Water purification system, ELGA PURELAB, (UHQ I, High Wycombe, United Kingdom) was utilized. Six-point calibration curve over the concentration range of 0.002–0.2 mM for RA standard solution with an *R*^2^ of 0.9951 was used for quantification of RA encapsulated amount. 

Phosphate buffer saline (PBS), pH 7.40, was chosen as a medium for in vitro drug release study. Precisely, 5 mg of both MI loaded beaded fibers, and control loaded beaded fibers were wetted with 2 mL PBS and placed in dialysis membrane pouch (regenerated cellulose, 0.64 in diameter, 14,000 molecular weight cut-off; Fisher Scientific Co., Hampton, NH, USA) dipped in 15 mL PBS. The release study was performed in a shaking water bath (GFL 1083; THERMOLAB^®^, Burgwedel, Germany) maintained at 37 °C at 100 rpm. At regular time intervals, aliquots of 0.8 mL were removed for analysis and replaced with the same volume of fresh PBS. The aliquots were characterized by HPLC at 330 nm. A six-point calibration curve over the concentration range of 0.001–0.2 mM for RA standard solution with an *R*^2^ of 0.9996 was utilized for quantification of the amount released, and the cumulative RA release percentage was calculated. In order to determine the release kinetics of RA from MI beaded fibers and control beaded fibers over time, the drug release data were fitted in the mathematical equations of first-order, zero-order, and Higuchi diffusion model. 

### 2.6. Magnetic Actuation System 

An orthogonal configuration of four electromagnetic coils is used to exert magnetic force on the MI beaded fibers. The fibers are contained in the common center of the coils inside a water reservoir. Four coils are independently powered and used to generate the controlled magnetic fields. Each electromagnetic coil (inner diameter 20 mm, outer diameter 40 mm, and length 80 mm) has 3200 turns and thickness of the wire is 0.7 mm, and it generates maximum magnetic field of 18 mT in the workspace using current input of 1 A [16]. This weak magnetic field does not yield any negative side effect and is lower than the field used in magnetic resonance imaging (MRI) by three orders of magnitude.

## 3. Results and Discussion

### 3.1. MI Fiber Preparation

PCL was selected as the supporting fiber matrix, as it is a biocompatible and biodegradable polymer. Furthermore, it has previously been used in micro- and nanofiber fabrication via electrospinning [28,29,30]. The concentration was considered among the most effective parameters for controlling the fiber morphology. PAM was used as a functional polymer for providing the molecular recognition binding sites. No phase separation was observed during the experiments. Upon complete solvent evaporation, molecular interactions between the amino groups of PAM and the carboxyl and phenol groups of RA are apparently dominant. PCL provided additional polymer interchain interactions for stabilizing the binding sites [31].

Tetrahydrofuran (THF) and dichloromethane (DCM) were examined as solvent systems at moderately low (7% *w*/*v*) PCL concentration, as useful electrospinnability is frequently achieved upon preparing PCL solutions in solvents of moderate-to-high solubility with likewise moderate dispersion forces [32]. Using DCM as solvent produced fragile fibers; the quality of those fibers was insufficient for magnetic control applications. Despite an improved quality of the beaded fibers using THF as solvent system, salting out effects were observed upon addition of RA–PAM mixture. Therefore, a binary solvent system with (1:1 *v*/*v*) mixture of both solvents was selected, which finally yielded fibers of acceptable quality enabling magnetic control. 

### 3.2. Design of Experiments Methodology

#### 3.2.1. Regression Analysis

DOE is a powerful experimental design technique that is capable of exploring the effects of significant factors and the optimal conditions for various manufacturing techniques [33]. A full factorial central composite design was adopted for the optimization of morphologies of magnetic electrospun fibers, where 4 factors, i.e., polymer concentration (4–16% *w*/*v*), collector distance (1–17 cm), flow rate (8–20 µL/min), and applied voltage (13–25 kV), were varied. SEM images collected for each run (shown in Supplementary Figure S1 and Figure S2) revealed randomly oriented fiber mats due to the use of simple static collecting surface during electrospinning. Fiber mats produced within the design limits had a wide range of fiber diameters with different morphological characteristics, as discussed later. 

Multiple regression analysis was performed using the results obtained to study and model the relationship between the four factors and each response variable. Equations (1)–(3) were generated for average FD, NB, and BD, respectively. For achieving a better data fit, values of results obtained were transformed using the natural logarithm:

ln FD = −4.18 + 0.7501 PC + 0.0662 CD + 0.3169 FR + 0.419 V − 0.03511 PC^2^ − 0.00396 CD^2^ − 0.01528 FR^2^ − 0.01090 V^2^ + 0.01077 PC × FR (1)

ln NB = 1.716 − 0.7876 PC(2)

ln BD = 7.405 + 0.0981 PC − 0.1708 CD + 0.01078 CD^2^ (3)

ANOVA analysis revealed that all terms obtained for the three regression models had *p*-values ≤ 0.05 indicating that the modeled terms are indeed significant. The obtained ln FD, ln NB, and ln BD values fitted the regression lines with percentages of 96.51%, 96.48%, and 58.96% (adjusted *R*^2^), respectively, whereas the ability to predict new observations was obtained at an accuracy of 93.66%, 95.82%, and 54.05% (predictive *R*^2^), respectively. Thus, ln BD regression model could not be used for response evaluation. Failure of obtaining a sufficiently adequate regression model to explain the effect of changing parameters on bead size reflects the inability to control the Taylor cone during electrospinning, which impedes controlling the bead size [18]. However, it was previously reported that BD is related to FD, where the smaller the FD, the smaller the BD and the shorter the distance between beads [13]. Furthermore, ANOVA analysis revealed a low standard error of regression (S) of 0.12 and 0.42 for ln FD and ln NB, respectively, where S indicates the average distance that the data points lie from the regression line. Residual plots (Supplementary Figure S3) are an alternative diagnostic tool that can evaluate the robustness of model fit and have revealed a normal distribution of the residuals. It is important to point out that there was not enough evidence that outliers exist in both models at a 5% significance level.

#### 3.2.2. Graphic Modeling 

**Figure 2:**
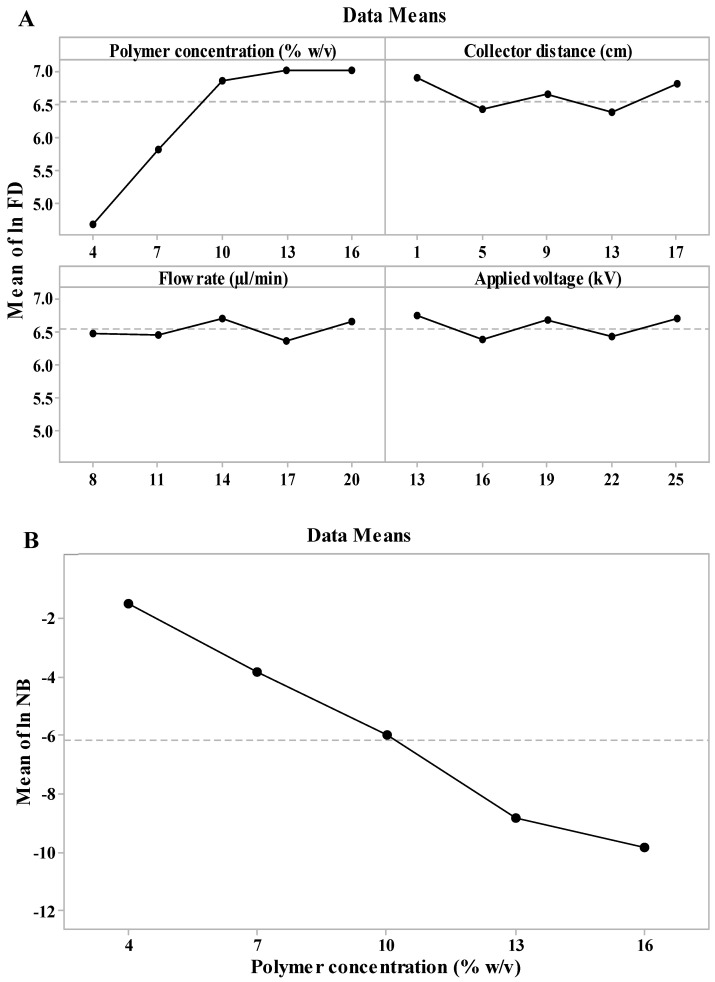
Main effect plots of (**A**) ln FD (fiber diameter) and (**B**) ln NB (number of beads per µm^2^) models.

Main effect plots were generated using the raw data response means to more easily visualize and compare the magnitudes of factors’ effect on the desired responses (Figure 2). It is clear from the main effect plots for the average FD (Figure 2A) that the polymer concentration had the most substantial influence on ln FD response, whereas Figure 2B displays the inversely linear relationship between polymer concentration and ln NB response.

The polymer concentration played the most crucial role during fiber formation within this study, whereby the changes in concentration led to the formation of electrospun mats of different morphologies, i.e., either beads, beaded fibers, or uniform fibers with different FD. The fiber morphology is mainly controlled by the charge carrying density, surface tension, and the viscoelasticity of the microemulsion being electrospun, which are factors mainly affected by the polymer concentration [34].

The PCL concentrations used in this study were in the range of 4–16% *w*/*v*. Figure 2A,B suggests that upon using low polymer concentration during electrospinning, the droplet surface charge is low, and the electrostatic field force is not capable of overcoming the gravitational force acting at the droplet and the surface tension, resulting in formation of a bead-like morphology. Moreover, lower concentrations enabled higher stretching of the polymer jet due to higher polymer chains mobility and higher jet instabilities, thus resulting in reduced fiber diameters. As the polymer concentration increased, the viscosity increased and the surface charge density and polymer chain entanglement in solution increased. As a result, the electrostatic field force is able to overcome gravitational forces acting upon the droplet and surface tension, and the jet ability to stretch is limited. This leads to a more uniform fiber morphology with increased FD. At the highest concentrations, further increase in FD is no longer observed, as the increase in viscosity and polymer chain entanglement renders the ejection of the polymer jet more difficult [34,35,36].

SEM images show the results of the 28 trials in Supplementary Figure S1 and Figure S2. Although axial point 19 prepared using the lowest PCL concentration of 4% *w*/*v* provided mostly beaded fiber morphology with very thin fiber diameters, axial point 20 with PCL concentration of 16% *w*/*v* yielded fully nonbeaded fiber mats with large fiber diameters. Moreover, all points prepared using a polymer concentration of 7% *w*/*v* resulted in beaded fiber mats with a high number of beads per µm^2^ (runs 1, 3, 5, 7, 9, 11, 13, and 15). Increasing the polymer concentration to 10% *w*/*v* caused the presence of fewer beads, (runs 17, 18, 21, 22, 23, 24, 25, 26, 27, and 28), and by reaching 13% *w*/*v*, almost no beaded fiber mats were produced (runs 2, 4, 6, 8, 10, 12, 14, and 16). 

Interaction plots (Supplementary Figure S4) were used to visualize the effect of interactions between polymer concentration and flow rate on ln FD response, by displaying the relation between one factor and the response depending on the values of the highest and lowest levels of the other factor. The nonparallel lines indicate the presence of significant interactions between the two factors as illustrated by the resulting *p*-value of 0.005. 

Contour plots (Supplementary Figure S5) graphically represent the relation between each two continuous variables and ln FD response, while holding the other variables constant. The lightest green regions represent the lowest transformed average FD values, whereas the darkest green regions represent the highest response values. It could be observed that ln FD is mainly responsive to changes in polymer concentration, whereby highest ln FD could be achieved using a combination of factors comprising high polymer concentrations. 

#### 3.2.3. Test of the Model Predictions

**Figure 3:**
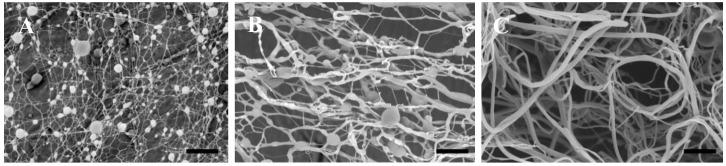
SEM images of the 3 prediction runs at 4000×, where the coordinates for Run (**A**) are (5, 8, 18, and 14), Run (**B**) are (8, 13, 15, and 24), and Run (**C**) are (14, 15, 10, and 18). Scale bar is 15 µm.

The predictive ability of the two regression models was tested by comparing the computed predictions for three electrospinning runs (factors settings see Figure 3), to the experimental data derived from the fabricated electrospun mats. The average FD and NB were calculated for each run using SEM images (Figure 3). Prediction interval and predicted fit for each run were generated at a 95% confidence level. All experimental results were within the predicted ranges, as shown in Table 1. This allows the conclusion that the regression models have adequate predictive ability for reliably optimizing the conditions for preparing magnetic beaded fibers with specific FDs and NBs.

### 3.3. Optimization of the Fiber Fabrication

The optimal conditions allowing magnetic motion control of MI beaded fibers that can act as drug reservoir with optimal controlled release kinetics were computed. The goal was to produce beaded fibers with possibly large FD (~1500 nm) and high NB (~0.0067 beads per µm^2^).

**Figure 4:**
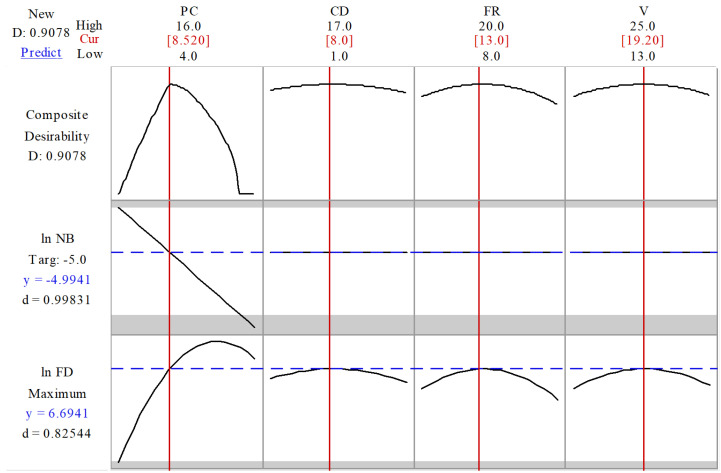
Optimization plot detecting the optimum factors settings to achieve beaded fibers with high FD and intermediately high NB, where the vertical redlines represent the factor setting chosen (its value is shown at the top of each column in red), whereas the horizontal blue lines represent the predicted response values (its value is shown on the left of each column in blue). Composite (D) and individual desirability (d) scores are displayed on the left.

Figure 4 illustrates the optimization plot, whereby each column represents a factor and the individual plots display the influence of each factor on the response. Individual desirability (d) of each response was computed to determine how well the combination of factor settings fulfills the goals of that response. The geometric mean of the two individual desirabilities (i.e., the composite desirability (D)) was computed to determine how well the combination of factor settings fulfills the goals of the combined responses. The desirability has a range with a minimum of zero and a maximum of one. Ideally, a composite desirability of one should be achieved, whereas a composite desirability of zero implies that one or more responses are not within their acceptable range. 

**Figure 5:**
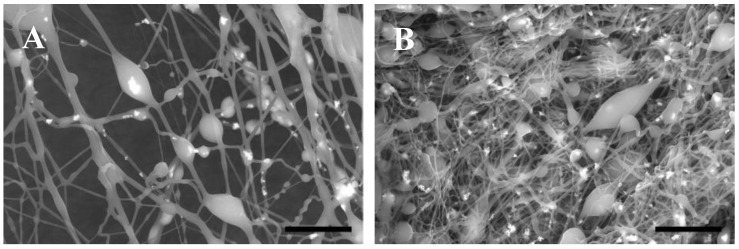
SEM images of (**A**) the optimized MI beaded fibers and (**B**) the control beaded fibers fabricated at 8000×. Scale bar is 10 µm.

The expected ln FD and ln NB values upon utilization of the computed optimized conditions, i.e., PCL concentration 8.52% *w*/*v*, collector distance 8 cm, flow rate 13 µL/min and applied voltage of 19.2 kV were 6.694 and −4.99, respectively, with a D of 0.9078. The experimental application of the optimized conditions yielded a beaded fiber mat with acceptable fiber diameters of ln FD and ln NB values at 6.75 and −4.51, respectively (Figure 5A). The experimental results were in agreement with the predicted responses with the predicted ranges of ln FD and ln NB at (6.41–6.98) and ((−5.89)–(−4.10)), respectively. Thus, the developed models are useful for reasonably accurate morphology predictions and fiber mats with maximum FD and high NB, as experimentally verified.

Control fibers were fabricated by directly incorporating RA into PCL solution following the same procedure except for the addition of PAM. The morphology is shown in Figure 5B. It was compared to the MI beaded fibers. It is evident that the incorporation of the RA–PAM complex had no noticeable impact on the bead formation, as confirmed with the NB of 0.011 ± 0.002 and 0.012 ± 0.002 per µm^2^ for MI and control beaded fibers, respectively. However, the absence of the complex has affected the FD, as the average FD of MI and control beaded fibers was 857 ± 390 and 334 ± 135 nm, respectively.

### 3.4. Characterization of Optimized MI Fibers

**Figure 6:**
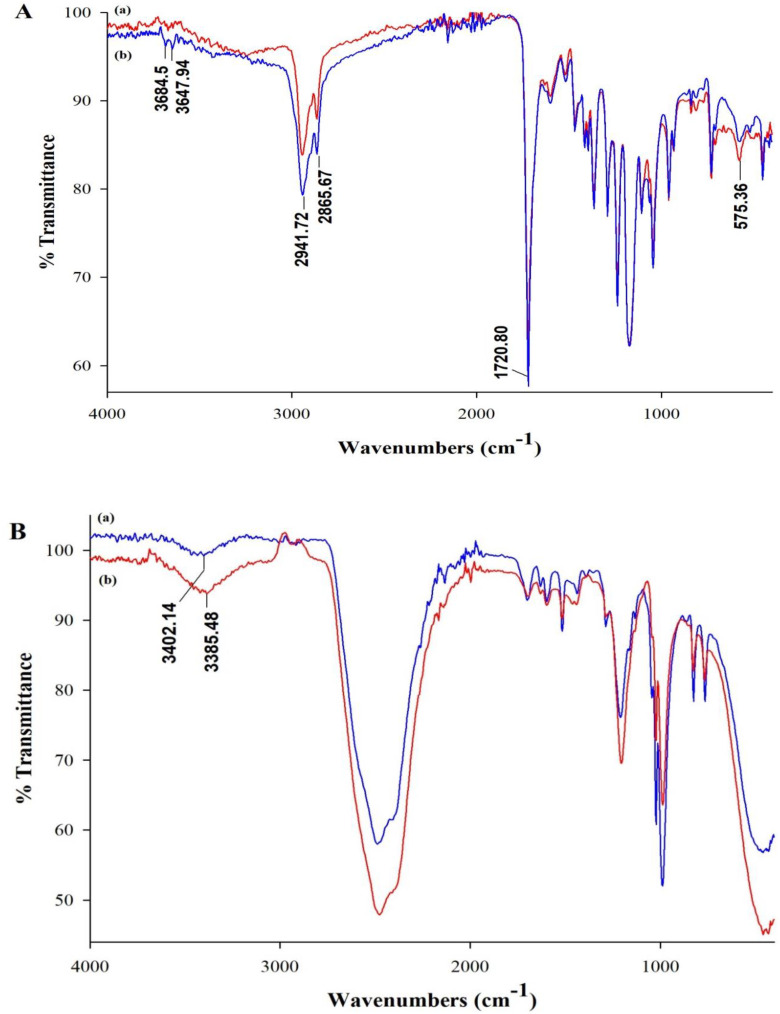
IR spectra of (a) control fibers and (b) MI fibers (**A**) and (a) pure RA and (b) RA–PAM complex in deuterated solvent (**B**).

The IR analysis revealed similarity between MI and control fibers via the obtained spectra, as shown in Figure 6A. The characteristic peaks of the PCL matrix are evident at 1721, 2866, and 2941 cm^−1^, which are assigned to C=O stretching of ester carbonyl group and symmetric and asymmetric CH_2_ stretching vibrations, respectively [37]. The vibrational peak at 575 cm^−1^ could be assigned to Fe-O stretching vibration of the added magnetite [38]. Additional peaks at 3648 and 3685 cm^−1^ present within the MI fiber spectrum correspond to the N–H stretching of PAM [39]. To confirm the assembly of RA with PAM, IR analysis of RA and RA–PAM complex solutions prepared using deuterium oxide was performed. Few drops of dimethyl sulfoxide-d6 were first added to ensure dissolution of RA. Figure 6B revealed a broader O–H stretching peak within the RA–PAM complex spectrum with a 16 cm^−1^ red shift of the peak at 3402 cm^−1^ present within the pure RA spectrum, thus indicating H-bond formation between the two species [40,41].

The results of the EDX analysis for MI fibers are presented in Table 2. The presence of nitrogen in both beads (heads) and fibers (tails) indicate that PAM resides in both parts. Thus, the formed fibers are composed of evenly blended structures of PCL and PAM, which reflects that the presence of RA is not exclusive to a specific part of the fabricated beaded fiber. It is also evident that the developed MI fibers are composed of a magnetic head, as iron is mainly located in the beads, and a flexible tail, which could provide a magnetic dipole moment and propulsion, respectively. This composition would perfectly allow the fabricated magnetic beaded fiber to act as a magneto sperm, which mimics the locomotion of a living sperm upon application of an external magnetic field.

### 3.5. In Vitro Release Studies

**Figure 7:**
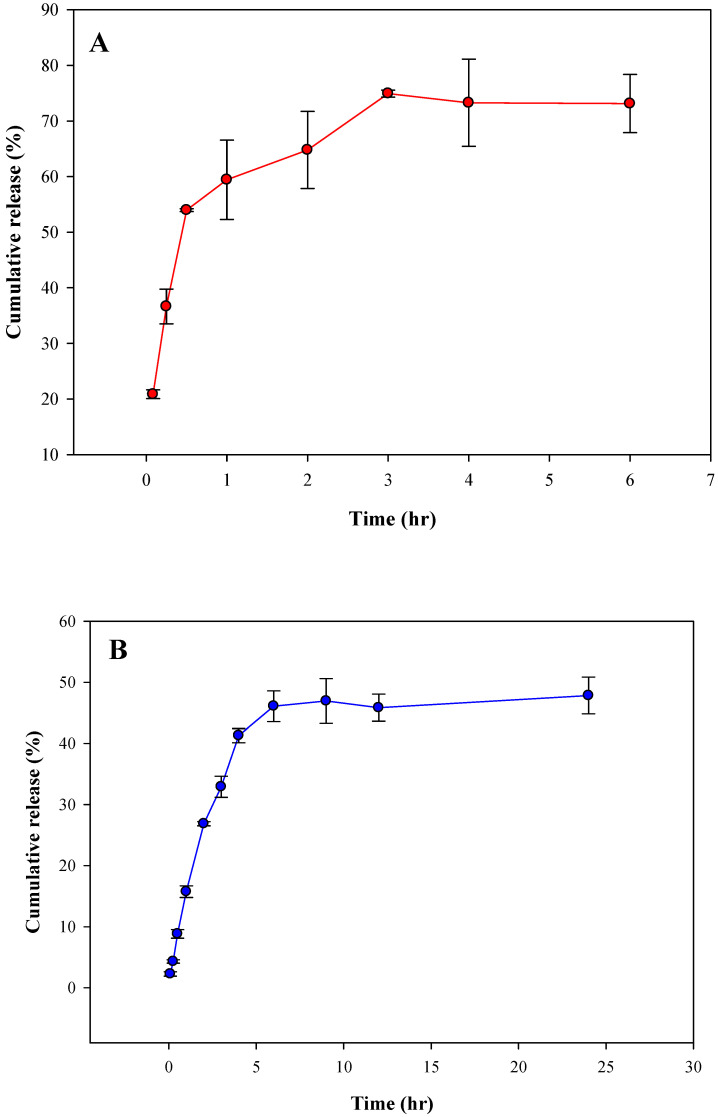
Cumulative percent release profile for (**A**) control beaded fibers over a period of 6 h and (**B**) MI beaded fibers over a period of 24 h.

RA encapsulated within MI beaded fibers was found to be 0.59 ± 0.01 mg/5 mg, whereas for control beaded fibers (not imprinted), an amount of 0.67 ± 0.03 mg/5 mg was achieved. It was expected that 5 mg of the control beaded fibers would contain more RA encapsulated since the matrix of the control beaded fibers did not include PAM. The in vitro release profile of RA in control and MI beaded fibers was studied in PBS for 6 h. Cumulative percentage release profile shown in Figure 7 revealed that MI beaded fibers were characterized by a much slower burst release vs. the control beaded fibers with initial releases of 2.3% ± 0.4% and 20.9% ± 0.8%, respectively. After the initial burst release, a slow and steady release was observed for both types of beaded fibers, whereby after 6 h, the cumulative released percentage was 46% ± 3% and 73% ± 5%, respectively.

The regression coefficient for RA release from MI beaded fibers was found to be 0.93, 0.96, and 0.99 for a zero-order, first-order, and Higuchi diffusion model, respectively. As for the control beaded fibers, *R*^2^ was 0.75, 0.87, and 0.9 for a zero-order, first-order, and Higuchi diffusion model, respectively. Drug release kinetics studies revealed that RA was released from both types of PCL beaded fibers by diffusion, as it gave the highest *R*^2^. However, the matrix composition and morphology apparently influenced the RA release rate. In addition to its larger FD, MI fibers apparently provided additional hydrogen bond interactions formed with RA due to the specific binding sites created by the amino group of PAM, which limited a faster release of RA. A cumulative released percentage of 48% ± 3% was achieved after 24 h, whereas for control fibers, the cumulative released was 73% ± 5% after 6 h already. These results indicate that magnetic MI beaded fibers have the potential to be used as vehicles for a more extended and sustained release of RA vs. nonimprinted fibers.

### 3.6. Magnetic Control of MI Sperm-Shaped Magnetic Beaded Fibers

Motion of the beaded fibers is governed by a force balance between the magnetic force and the hydrodynamic drag on the head and the tail. The fibers are pulled by a magnetic force while immersed in a medium characterized by a low Reynolds number ($Re=\frac{\rho UL}{\mu }$). The hydrodynamics are on the order of $O\left(10^{-3}\right)$, where *ρ* and *µ* are the density and viscosity of the medium, respectively. Furthermore, L and U are the length of the fiber and its forward velocity, respectively. The relation between the drag force and the velocity of the fiber is linear and is balanced by the magnetic forces exerted on its magnetic dipole moment as follows: $\left(\nabla \cdot m\right)B+F_{h}+F_{t}=0$, where m is the magnetic dipole moment of the beaded fiber and B is the external magnetic field [17,42]. Furthermore, $F_{h}$ and $F_{t}$ are the drag forces on the head and tail of the beaded fiber, respectively. The first term in the force balance represents the control input and is determined based on the desired position vs. the measured position of the beaded fiber.

**Figure 8:**
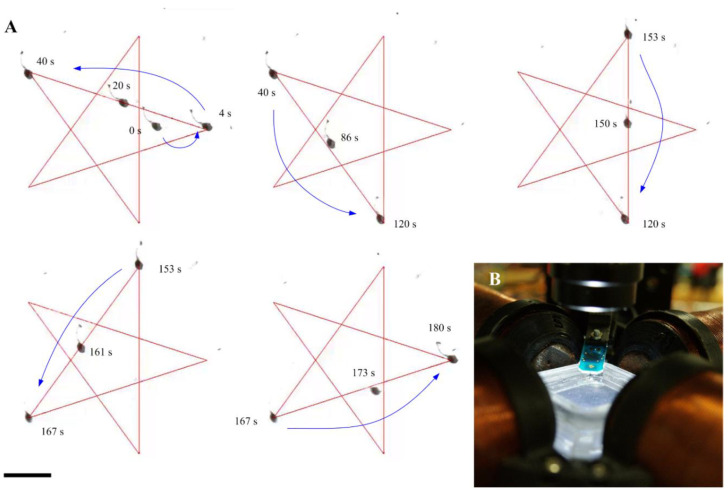
Motion control of a magnetic MI beaded fiber is achieved using controlled magnetic field gradients. (**A**) The beaded fiber is pulled along a star-shaped trajectory by a controlled magnetic force. The blue arrow indicates the direction of the beaded fiber. (**B**) The magnetic field gradient is generated using 4 electromagnetic coils. The beaded fiber is contained inside a water reservoir in the common center of the 4 coils. Motion of the beaded fiber is tracked using 3X Mitutoyo phase objective. Scale bar is 100 µm.

Figure 8 shows a representative closed-loop motion control experiment of a MI beaded fiber with length of 100 µm. The beaded fiber is pulled toward waypoints along the star-shaped predefined trajectory shown in Figure 8A. The position error (e) is determined based on the position of the beaded fiber and the waypoints along the star-shaped trajectory. This error is used to determine the magnetic field of the magnetic system by setting, $\left(\nabla \cdot m\right)B=k_{p}e$+$k_{d}\dot{e}$, where $k_{p}$ and $k_{d}$ are positive-definite gain matrices and $\dot{e}$ is the velocity tracking error. This closed-loop control achieves a maximum tracking error of 20 µm with an average velocity of the beaded fiber measured as 40 ± 20 µm/s.

## 4. Conclusions

The present study proposed a simple, robust, and reliable method for the fabrication of molecularly imprinted biocompatible nanofibers using electrospinning. Central composite design was used to optimize the main factors with a reported effect on the morphology of electrospun fibers, i.e., the polymer concentration, the collector distance, the flow rate, and the applied voltage. The generated mathematical models were able to correlate the changes in fiber morphology (i.e., fiber diameter and number of beads per µm^2^) to these factors, and an optimum polymer concentration was determined as the crucial parameter. Thus, determined optimal factor settings were then applied to experimentally obtain electrospun beaded fibers with fiber diameters of 857 ± 390 nm and 0.011 ± 0.002 beads per µm^2^ at a polymer concentration of 8.52% (*w*/*v*), a collector distance of 8 cm, a flow rate of 13 µL/min, and an applied voltage of 19.2 kV. Moreover, the obtained results revealed that magnetic beaded fibers may indeed be propelled in a highly controllable fashion under the influence of externally generated magnetic field gradients along a predefined trajectory with a maximum tracking error of 20 µm. Along with controllable sustained drug release properties, as shown for molecularly imprinted fibers, such microrobots have been verified as a promising future tool for targeted drug delivery systems.

## Figures and Tables

**Table 1:** Experimental and predicted ln fiber diameter (FD) and ln number of beads present per µm^2^ (NB) values for three experiments.

Run	ln FD			ln NB		
	Experimental	Predicted	Prediction Range	Experimental	Predicted	Prediction Range
**A**	4.64	4.42	4.05–4.79	−2.98	−2.22	(−3.16)–(−1.28)
**B**	6.33	6.12	5.81–6.44	−5.01	−4.58	(−5.48)–(−3.69)
**C**	6.88	6.70	6.37–7.03	−9.00	−9.31	(−10.23)–(−8.39)

**Table 2:** Chemical composition of molecularly imprinted (MI) beaded fibers (in atom %).

Sample	C %	N %	O %	Fe %
**Head**	70.21	4.47	23.65	1.67
**Tail**	74.51	3.95	21.53	-

## Supplementary Materials

The following are available online at https://www.mdpi.com/2079-4991/10/8/1478/s1, Table S1: central composite design experimental runs settings and average response values; Figure S1: SEM images of runs (1–16) of central composite experimental design at 4000×; Figure S2: SEM images of runs (17–28) of central composite experimental design at 4000×; Figure S3: residual plots of ln fiber diameter and number of beads per µm^2^; Figure S4: interaction plot for ln fiber diameter; and Figure S5: contour plots for ln fiber diameter.
