# Supplementary material for: Fabrication of Magnetic Molecularly Imprinted Beaded Fibers for Rosmarinic Acid
Source: Nanomaterials (Basel). 2020 Jul 28;10(8):1478. doi: 10.3390/nano10081478 (PMC7466393; doi:10.3390/nano10081478)
Supplement: Supplementary file 1 [file nanomaterials-10-01478-s001.pdf]

## Supplementary Materials

# Fabrication of Magnetic Molecularly Imprinted Beaded Fibers for Rosmarinic Acid

**Engy M. Saad [1], Nesrine Abdelrehim El Gohary [1,*], Basma M. El-Shenawy [2], Heba Handoussa [3], Anke Klingner [4], Mohamed Elwi [5], Youssef Hamed [6], Islam S. M. Khalil [7], Rasha Mohamed El Nashar [8,*] and Boris Mizaikoff [9,*]**

1 Pharmaceutical Chemistry Department, Faculty of Pharmacy and Biotechnology, German University in Cairo, Cairo 11835, Egypt; Engy.yehia@guc.edu.eg
2 Pharmaceutical Technology Department, Faculty of Pharmacy and Biotechnology, German University in Cairo, Cairo 11835, Egypt; basma.el-shenawy@guc.edu.eg
3 Pharmaceutical Biology Department, Faculty of Pharmacy and Biotechnology, German University in Cairo, Cairo 11835, Egypt; heba.handoussa@guc.edu.eg
4 Physics Department, German University in Cairo, Cairo 11835, Egypt; anke.klingner@guc.edu.eg
5 Materials Engineering Department, Faculty of engineering and Materials Science, German University in Cairo, Cairo 11835, Egypt; mohamed.elwi@guc.edu.eg
6 Mechatronics Department, Faculty of engineering and Materials Science, German University in Cairo, Cairo 11835, Egypt; youssef.sabry@student.guc.edu.eg
7 Department of Biomechanical Engineering, University of Twente, 7522 NB Enschede, The Netherlands; i.s.m.khalil@utwente.nl
8 Chemistry Department, Faculty of Science, Cairo University, Giza 12613, Egypt
9 Institute of Analytical and Bioanalytical Chemistry, Ulm University, 89081 Ulm, Germany
* Correspondence: nesrine.elgohary@guc.edu.eg (N.A.E.G.); rashaelnashar@gmail.com (R.E.N.); Boris.mizaikoff@uni-ulm.de (B.M); Tel.: +20-2-25412990 (N.A.E.G.); +49-731-50-22750 (B.M.); +20-01005044622 (R.E.N.)

Received: 1 July 2020; Accepted: 25 July 2020; Published: date

**Table 1.** Central composite design experimental runs settings and average response values.

| Run | PC (% *w*/*v*) | CD (cm) | FR (μL/min) | V (kV) | FD (nm) | NB (/μm²) | BD (nm) |
|---|---|---|---|---|---|---|---|
| **1** | 7 | 5 | 11 | 16 | 412.48 | 0.023500 | 2087.21 |
| **2** | 13 | 5 | 11 | 16 | 1162.23 | 0.000202 | 3848.06 |
| **3** | 7 | 13 | 11 | 16 | 302.38 | 0.029200 | 1791.15 |
| **4** | 13 | 13 | 11 | 16 | 890.32 | 0.000247 | 4010.36 |
| **5** | 7 | 5 | 17 | 16 | 258.41 | 0.028100 | 1698.72 |
| **6** | 13 | 5 | 17 | 16 | 1168.80 | 0.000139 | 2960.00 |
| **7** | 7 | 13 | 17 | 16 | 339.87 | 0.012200 | 2287.52 |
| **8** | 13 | 13 | 17 | 16 | 1201.42 | 0.000105 | 3163.56 |
| **9** | 7 | 5 | 11 | 22 | 454.47 | 0.021000 | 1733.38 |
| **10** | 13 | 5 | 11 | 22 | 1012.75 | 0.000097 | 4250.44 |
| **11** | 7 | 13 | 11 | 22 | 381.65 | 0.021500 | 2210.27 |
| **12** | 13 | 13 | 11 | 22 | 1174.42 | 0.000139 | 3249.67 |
| **13** | 7 | 5 | 17 | 22 | 301.11 | 0.023300 | 2106.76 |
| **14** | 13 | 5 | 17 | 22 | 1153.49 | 0.000173 | 1677.14 |
| **15** | 7 | 13 | 17 | 22 | 277.07 | 0.019700 | 1994.42 |
| **16** | 13 | 13 | 17 | 22 | 1213.98 | 0.000123 | 6823.00 |
| **17** | 10 | 9 | 14 | 19 | 1235.40 | 0.002220 | 1850.89 |
| **18** | 10 | 9 | 14 | 19 | 1114.84 | 0.002150 | 1748.96 |
| **19** | 4 | 9 | 14 | 19 | 108.19 | 0.226000 | 1474.11 |
| **20** | 16 | 9 | 14 | 19 | 1121.70 | 0.000053 | 5104.77 |
| **21** | 10 | 1 | 14 | 19 | 993.07 | 0.001600 | 3969.78 |
| **22** | 10 | 17 | 14 | 19 | 922.02 | 0.003960 | 5487.46 |
| **23** | 10 | 9 | 8 | 19 | 645.52 | 0.004440 | 2358.74 |
| **24** | 10 | 9 | 20 | 19 | 783.77 | 0.003540 | 3185.27 |
| **25** | 10 | 9 | 14 | 13 | 846.82 | 0.003060 | 1279.29 |
| **26** | 10 | 9 | 14 | 25 | 819.10 | 0.001390 | 2592.83 |
| **27** | 10 | 9 | 14 | 19 | 1220.77 | 0.002430 | 2299.21 |
| **28** | 10 | 9 | 14 | 19 | 1092.28 | 0.002080 | 3066.58 |

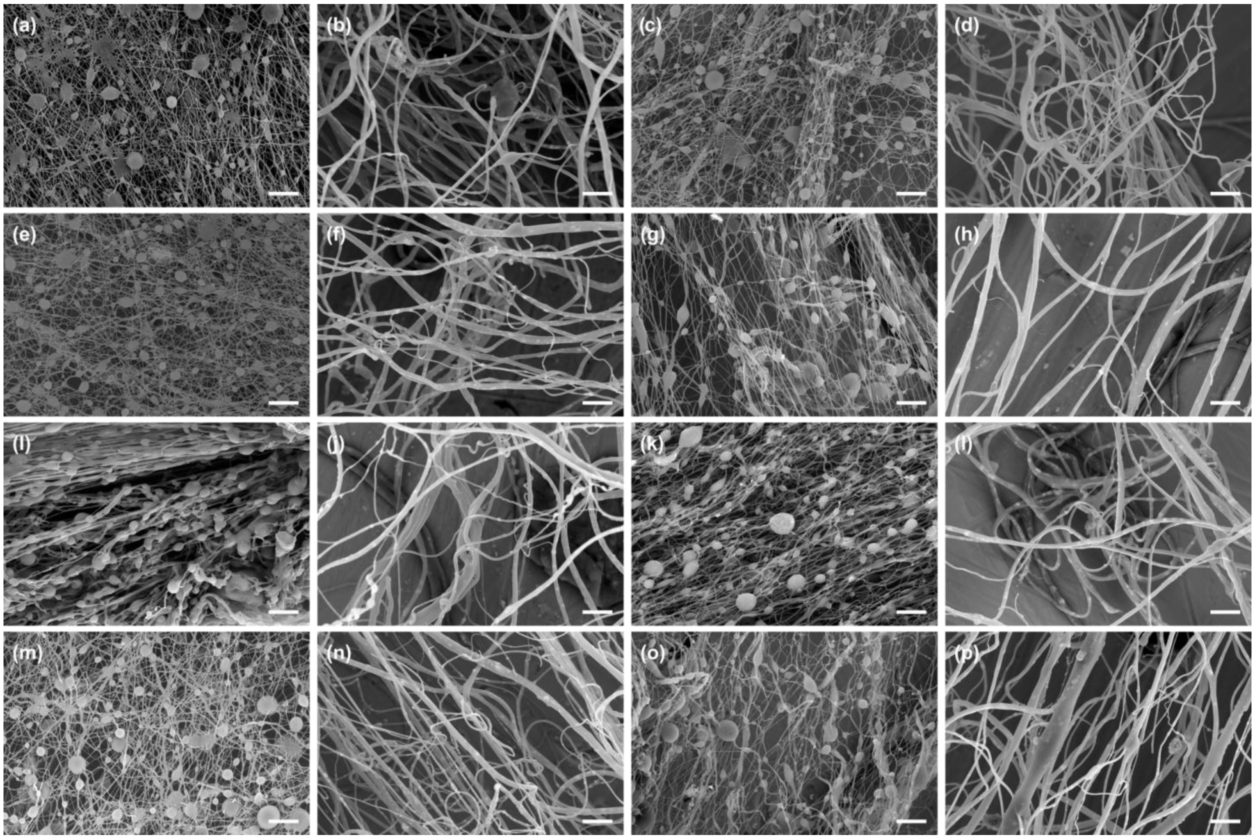


**Figure S1**: SEM images of runs (1–16) of central composite experimental design at 4000×. (**a**) cube point 1, (**b**) cube point 2, (**c**) cube point 3, (**d**) cube point 4, (**e**) cube point 5, (**f**) cube point 6, (**g**) cube point 7, (**h**) cube point 8, (**i**) cube point 9, (**j**) cube point 10, (**k**) cube point 11, (**l**) cube point 12, (**m**) cube point 13, (**n**) cube point 14, (**o**) cube point 15, (**p**) cube point 16. Scale bar is 10 um.

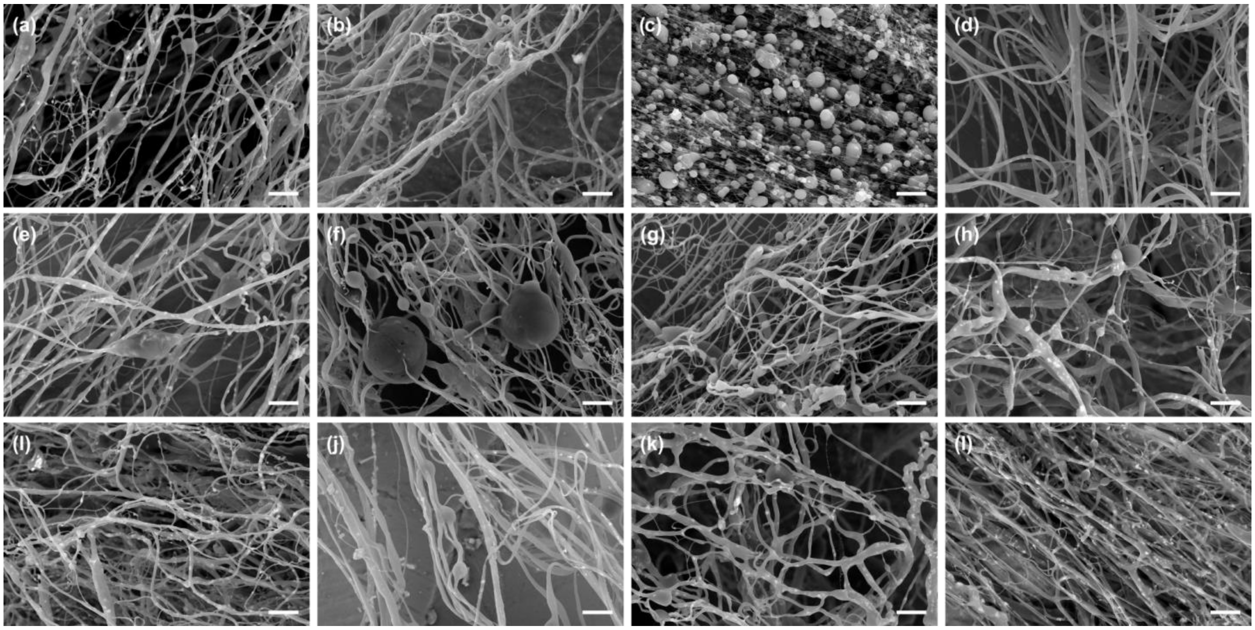


**Figure S2:** SEM images of runs (17–28) of central composite experimental design at 4000×. (**a**) center point 17, (**b**) center point 18, (**c**) axial point 19, (**d**) axial point 20, (**e**) axial point 21, (**f**) axial point 22, (**g**) axial point 23, (**h**) axial point 24, (**i**) axial point 25, (**j**) axial point 26, (**k**) center point 27, (**l**) center point 28. Scale bar is 10 um.

(**A**)

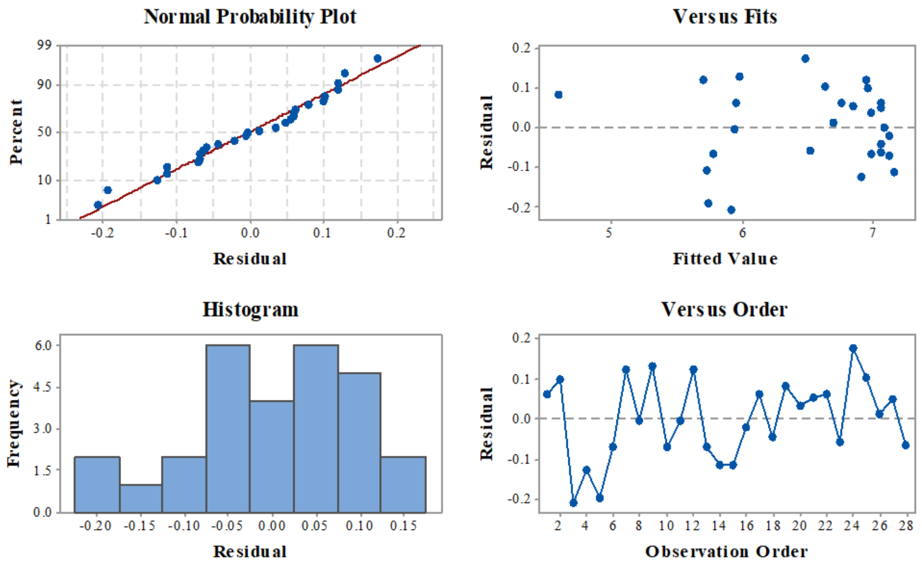


(**B**)

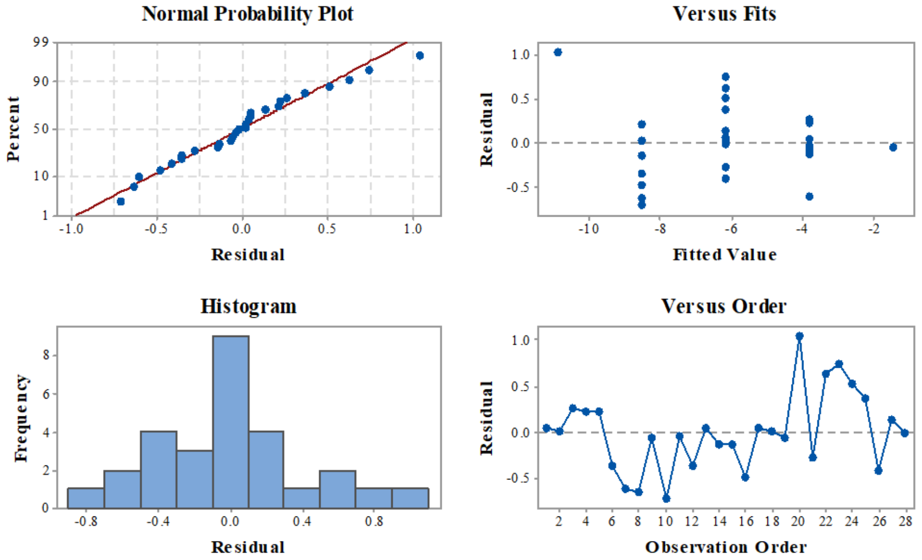


**Figure S3:** Residual plots of (**A**) ln FD and (**B**) ln NB models.

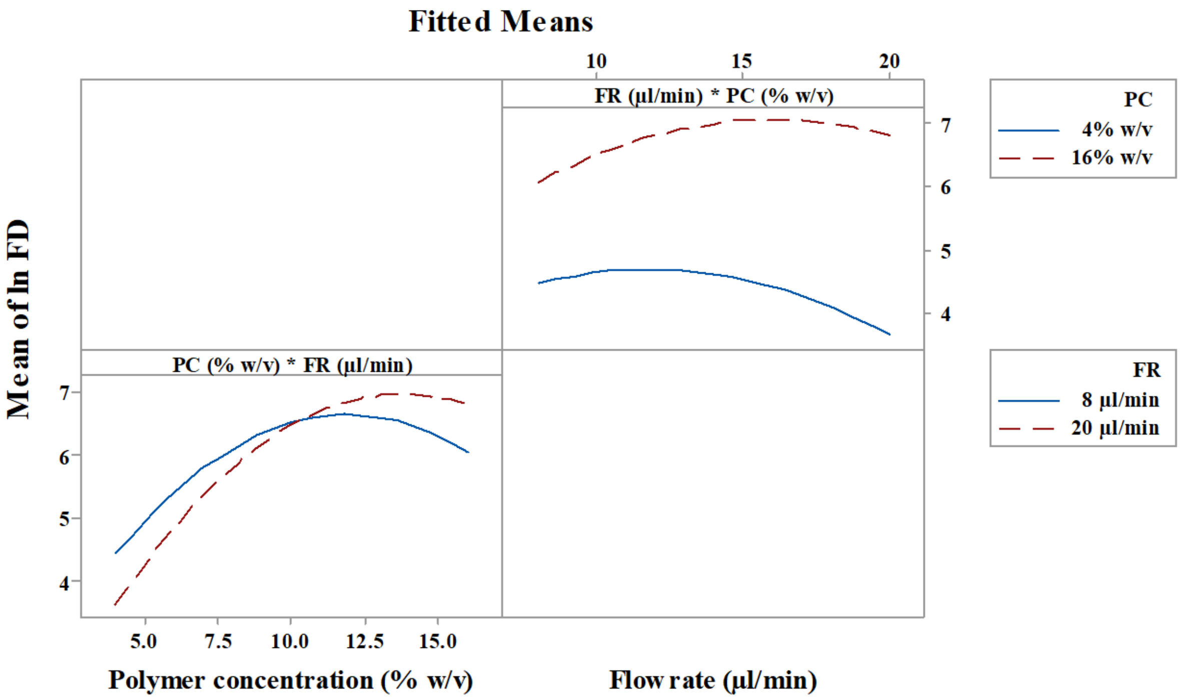


**Figure S4:** Interaction plot for ln FD.

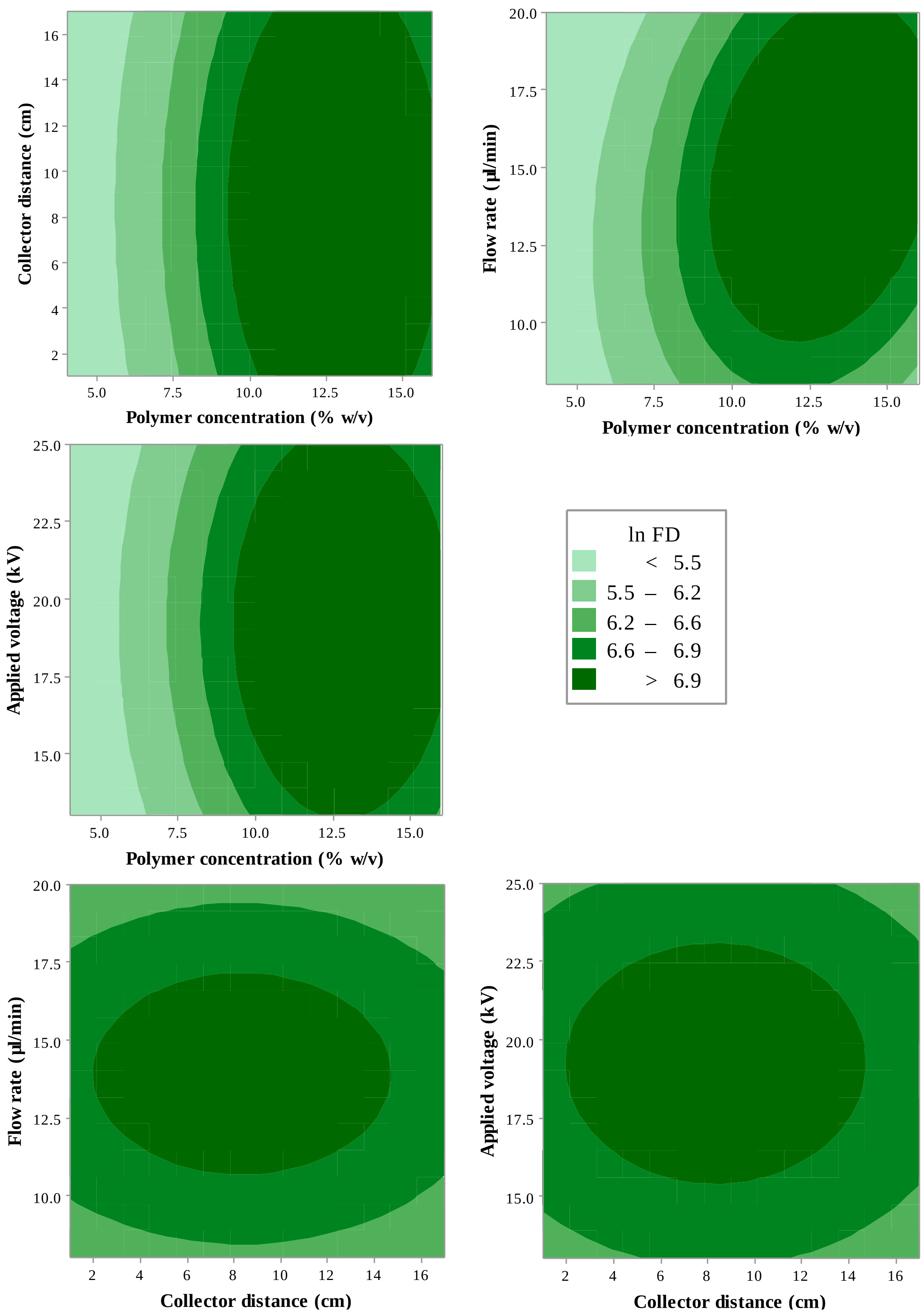

Collector distance (cm)
Polymer concentration (% w/v)
Flow rate (µl/min)
Applied voltage (kV)
ln FD
< 5.5
5.5 – 6.2
6.2 – 6.6
6.6 – 6.9
> 6.9

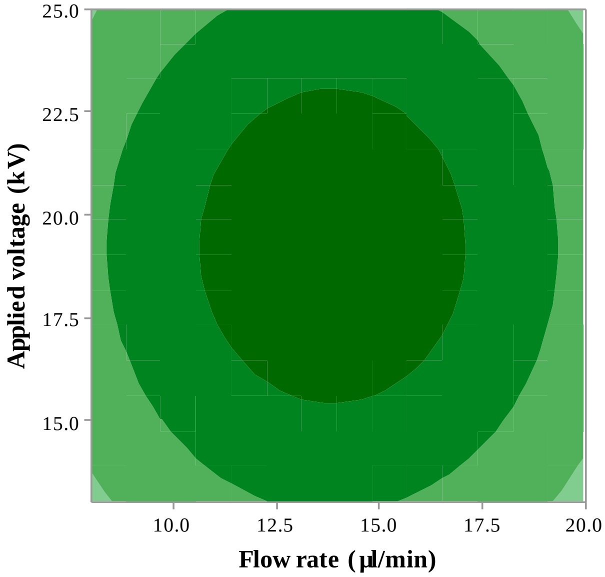


**Figure S5:** Contour plots for ln FD. The hold values are 10% *w*/*v* polymer concentration, 9 cm collector distance, 14 µL/min flow rate, and 19 kV applied voltage.
